# Effectiveness of Father-Focused Interventions to Prevent or Reduce Intimate Partner Violence During Pregnancy and Early Parenthood: A Systematic Review

**DOI:** 10.1177/15248380241277270

**Published:** 2024-09-20

**Authors:** Karen Wynter, Lauren M. Francis, Ashlee Borgkvist, Barnaby Dixson, Levita D’Souza, Elisabeth Duursma, Chris May, Louisa Sher, Jennifer St George

**Affiliations:** 1 Department of Psychiatry, School of Clinical Sciences, Monash University, Australia; 2 School of Nursing & Midwifery, Deakin University, Australia; 3 Centre for Social and Early Emotional Development, School of Psychology, Deakin University, Australia; 4 Safe Relationships and Communities Research Group, University of South Australia, Australia; 5 School of Health, University of the Sunshine Coast, Australia; 6 School of Psychology, The University of Queensland, Australia; 7 School of Educational Psychology and Counselling, Monash University, Australia; 8 Transforming early Education And Child Health (TeEACH) Research Centre, School of Education, University of Western Sydney, Australia; 9 School of Education, University of Wollongong, Australia; 10 School of Health Sciences, University of Newcastle, Australia; 11 Library, Deakin University, Australia

**Keywords:** batterers, domestic violence, intervention/treatment, cultural contexts

## Abstract

During pregnancy and the early parenting period, women are especially vulnerable to intimate partner violence (IPV), with devastating impacts on women, children, and families. The aim of this systematic review was to determine the effectiveness of father-focused interventions to prevent or reduce IPV during pregnancy and early parenthood. Six databases were searched, using a combination of the concepts “fathers,” “pregnancy/early parenthood,” “IPV” and “intervention.” Articles were double screened by title and abstract, and then full-text. Methodological and reporting quality was assessed using the Quality Assessment with Diverse Studies tool. Fifteen papers were eligible for inclusion; these articles were mostly of poor-to-moderate quality. Only three of the articles reported on interventions in lower- and middle-income countries. The most common forms of IPV addressed in these interventions were physical (10), psychological (8), sexual (4), and economic/financial (3). Of 12 articles reporting on data from both intervention and control groups, only six indicated statistically significant results; among these, only three reported robust analyses showing significantly greater reduction in IPV in intervention than in control groups. All three took place in lower- or middle-income countries. Two were underpinned by theoretical frameworks, which considered transforming traditional perceived gender norms. Therefore, interventions based on principles that address transformation of gender norms show promise but the success of such underlying principles needs to be confirmed, and better-quality evidence and reporting are needed for interventions targeting fathers to prevent or reduce IPV.

Intimate partner violence (IPV) is defined as “any act or behaviour within a present or former intimate relationship that causes physical, psychological, or sexual harm” (WHO, 2012). It includes acts of physical, sexual, and psychological violence as well as controlling behaviors (e.g., coercion, restricting access to financial resources, threats and intimidation, isolation, as well as abuse of children) (WHO, 2012). Although all genders may be subjected to IPV, it is widely recognized as a gendered societal issue, where IPV is most often perpetrated by men against women (Downes et al., 2019). A recent meta-analysis of the global prevalence of IPV (*N* *=* 366 studies) estimated that 27% of women aged 15 to 49 who had ever had a partner, had experienced physical and/or sexual IPV during their lifetime, and 13% had experienced IPV during the past year (Sardinha et al., 2022).

IPV against women can occur at all stages of life; however, the prevalence of IPV is reported to be higher among women of reproductive age than at other stages of life (Hasstedt & Rowan, 2016; Sardinha et al., 2022). During pregnancy specifically, the prevalence of any IPV (physical, psychological, or sexual violence) is estimated at 25% (Román-Gálvez et al., 2021). More than 10.3 million women in the United States (US) have had a male partner who attempted to get them pregnant against their will or refused to wear a condom, while 2.1 million women in the U.S. have become pregnant as a result of intimate partner rape (Hasstedt & Rowan, 2016). No clear estimates of the prevalence of IPV have been reported during the postpartum period and fewer studies have examined IPV during this period (Mojahed et al., 2021). Psychological violence has been found to be more prevalent than other forms of IPV during the perinatal period (pregnancy and the postnatal period) (Mojahed et al., 2021). Women may therefore be more vulnerable to all forms of IPV during the perinatal period than at any other stage of life.

## Impact of IPV on Mother and Child

IPV during the perinatal period can negatively impact mother and child health outcomes (Finnbogadóttir et al., 2020; Pastor-Moreno et al., 2020). Sexual IPV is associated with mothers’ late entry into prenatal care (Romero-Gutiérrez et al., 2011) and low infant birthweight (Khaironisak et al., 2017). Exposure to any IPV is associated with a significantly higher risk of symptoms of postnatal depression among women (Ankerstjerne et al., 2022). In addition, exposure to violence in pregnancy has been associated with poorer attachment relationships between mother and fetus (Zare et al., 2022).

In households where IPV against women occurs, young children are commonly exposed to this violence (Wolbers et al., 2023); these children are then at increased risk of subsequent physical, emotional, and sexual abuse (Holt et al., 2008; Savopoulos et al., 2022), emotional and behavioral problems, as well as compromised mental health, social and academic functioning, cognitive development (Bogat et al., 2023; Vu et al., 2016) and poor physical health and sleep (El-Sheikh et al., 2006). Negative physical and mental health outcomes from childhood exposure to IPV affect every period of childhood (Carlson et al., 2018), and may endure through to adulthood (Russell et al., 2010). In addition, child exposure to IPV is significantly associated with subsequent perpetration of IPV in adulthood (Kimber et al., 2018).

## Models of Violence Reduction

Systematic reviews of studies reporting effectiveness of interventions to reduce IPV across the lifespan demonstrate that most interventions focus on female victims (Prosman et al., 2015; Van Parys et al., 2014). While keeping women and children safe and together is a priority, interventions targeting women and children who experience violence can have a limited effect on reducing violence without targeting the male perpetrators. Intervening with men who perpetrate violence is therefore essential to reduce risk to women and children (Heward-Belle et al., 2019; Humphreys et al., 2020; Idriss-Wheeler et al., 2021). However, relatively few studies have reported specifically on interventions for men who perpetrate IPV (Idriss-Wheeler et al., 2021).

Models of violence reduction targeting men need to address a complex interaction between risk factors that have been associated with the perpetration of violence; these risk factors operate at individual, partner relationship, community, and social levels (Capaldi et al., 2012; Clare et al., 2021). Men’s younger age, lower education, poverty or food instability, unemployment, alcohol and other substance abuse, poor mental health, and relationship conflict have been found to be associated with perpetration of IPV by men (Capaldi et al., 2012; Clare et al., 2021). An association has also been found between men who have experienced or witnessed abuse or neglect during their childhood and increased likelihood of IPV perpetration during adulthood (Capaldi et al., 2012; Clare et al., 2021). Intervention programs need to take account of these individual-level risk factors associated with IPV perpetration (Gibbs, Dunkle, Ramsoomar et al., 2020; WHO & UNICEF, 2022).

Men’s beliefs and attitudes about the acceptability of the use of violence against and exertion of control over an intimate partner are strongly influenced by societal and cultural norms related to gender roles and ideals, but also by the norms men observe in their immediate community (Gibbs, Dunkle, Ramsoomar et al., 2020; Jewkes et al., 2020). Lack of, or inequal, social and economic rights among women compared to men, is a known risk factor for IPV (WHO, 2012). The World Health Organization (WHO) therefore recommends that prevention of IPV requires laws and policies to support gender equality and women’s social and economic rights (WHO, 2012). Thus, interventions to prevent or reduce IPV need to be guided by a theory of change that considers multiple risk factors associated with IPV relevant to specific contexts (Jewkes et al., 2021).

Programs addressing IPV may be primary or secondary prevention approaches. Primary prevention approaches aim to stop IPV before it occurs by targeting all participants, not only those who have perpetrated (men) or experienced (women) IPV (Graham et al., 2019). A recent multilevel meta-analysis of IPV primary prevention interventions showed a significant reduction in intervention participants’ risk of perpetrating or experiencing IPV (Alsina et al., 2023). Community-level and group-based primary interventions that aim to address gendered inequities have been shown to significantly reduce IPV against women in low- and middle-income countries, where groups may include men, women, or couples (Jewkes et al., 2020; Leight et al., 2023). A comprehensive report (Kerr-Wilson et al., 2020) concluded that when designed and executed well, specific types of IPV interventions are effective. These include economic transfer programs with or without social empowerment programs targeting women; parenting programs to prevent IPV and child maltreatment; community activism to address harmful gender attitudes, roles, and social norms; couple interventions to transform gender relations, with or without addressing alcohol and substance abuse; and school-based interventions. The authors report conflicting evidence about working with men or boys alone (Kerr-Wilson et al., 2020). Similarly, a recent systematic review concluded that there is no consistent evidence about “what works” for IPV perpetration prevention programs for boys and men (Graham et al., 2019). However, interventions including men, have been found to be more effective than interventions for women only (Alsina et al., 2023).

IPV secondary prevention programs aim to reduce existing IPV, targeting men who perpetrate violent IPV, and/or women who have experienced IPV (Graham et al., 2019), and include court-mandated programs (Hardesty & Ogolsky, 2020). Intervention formats for male perpetrators include group-based programs and individual case management (ANROWS, 2020b; Oliffe et al., 2022). Psychoeducational, psychological, and/or cognitive behavioral components are commonly employed to address behaviors employed by male perpetrators to exert fear and control over women (Chung et al., 2020; Gondolf, 2007; Mackay et al., 2015). Voluntary attendance at secondary prevention interventions is uncommon and attrition has been found to be high when interventions are not mandatory (McGinn et al., 2020). The difficulties that many services experience in recruiting and working with men complicate the understanding of perpetrator needs and the subsequent tailoring of programs (Cannon et al., 2016), ultimately impacting on assessment of program effectiveness (Augusta-Scott & Dankwort, 2002; Brown & James, 2014).

Overall, reviews and meta-analyses of interventions targeting men to prevent or reduce IPV at all life stages have shown mixed and weak evidence as to their effectiveness (Alsina et al., 2023; Cheng et al., 2021; Wilson et al., 2021; Yakeley, 2022). Studies most commonly report process outcomes (e.g., the number of participants attending a program) and participant feedback about program acceptability and feasibility. Authors report that there is little consistency in how studies are evaluated while noting substantial variation in the content, focus, facilitation, and setting of programs (Alsina et al., 2023; Babcock, Green, Webb et al., 2004; Babcock et al., 2016; Brown & James, 2014; Koehler et al., 2013). All of these factors make comparisons and conclusions difficult to draw (Babcock, Green, Robie et al., 2004; Hamilton et al., 2013; Lilley-Walker et al., 2018). Owing to the importance of targeting men in IPV interventions, further attempts to synthesize the evidence are important to inform future interventions and service delivery.

## Transition to Fatherhood: An Opportunity for Reduction and Prevention of IPV

The WHO recommends involvement of men in maternal and infant care (WHO, 2015), one of the benefits being that the transition to fatherhood presents an opportunity to support the learning of positive relationship skills and shared decision-making between men and their partners and children (Comrie-Thomson et al., 2021; Cooper et al., 2013; Labarre et al., 2016; Tokhi et al., 2018; WHO & UNICEF, 2022). Learning these skills may contribute to prevention or reduction in men’s perpetration of IPV (Capaldi et al., 2012; Clare et al., 2021). Even though it can be a time of stress and competing challenges, for men the transition to parenthood is often a period of identity reappraisal (Baldwin et al., 2018; Chin et al., 2011; Shorey & Ang, 2019). Although this transition period may also be a time of increased IPV perpetration (James et al., 2013) and fathers may view violence in distinct isolation from fatherhood (Veteläinen et al., 2013), many fathers may acknowledge the consequences of violence on their children and be motivated to change their behavior for the benefit of their children’s well-being (Oliffe et al., 2022). Programs can therefore aim to bridge the gap between men’s role as a father and their identity as someone who perpetrates violence (Stanley et al., 2012).

Not only is the transition to fatherhood an opportunity to intervene and encourage positive behavior change in men who perpetrate violence but also for a number of reasons this may be a critical window to *prevent* IPV before violent behaviors develop (Flynn, 2012). First, becoming a father is an opening for men to reevaluate their perceptions of gender norms and beliefs, and engage positively with their partners and children (Cooper et al., 2013; Oliffe et al., 2022). Second, pregnancy and the early parenting period represent a life stage when most families, including fathers, are in regular contact with health care providers (Johnston et al., 2015; Van Parys et al., 2014). Finally, universal psychoeducational interventions which include both mothers and fathers may well assist parents with infant settling (Fisher et al., 2010). Such interventions are important as anger and violence have been associated with unsettled infant behavior among fathers (Ellett et al., 2009; Long & Johnson, 2001; Reijneveld et al., 2004), who are known to experience poor sleep and enduring fatigue in the first year after a baby is born (Wynter et al., 2020). Indeed, effective inclusion of fathers in perinatal health services is recommended, including among families at risk of violence (Wynter et al., 2024).

Although the transition to fatherhood is a critical period in which interventions can contribute to the reduction or prevention of violence, a lack of research on the development and evaluation of strategies to prevent men’s perpetration of IPV during the transition to fatherhood has been reported (Sinnott & Artz, 2016). Researchers and advocates for women’s and children’s safety are calling for more rigorous, formal evaluations of interventions with fathers (ANROWS, 2020b; D’Inverno et al., 2018; Heward-Belle et al., 2019; Stanley & Humphreys, 2017) and for documentation of longitudinal and broader family outcomes (Oliffe et al., 2022).

The aim of this systematic review was to investigate the effectiveness of father-focused interventions to prevent or reduce IPV during pregnancy and early parenthood.

## Methods

The review method and reporting followed the Preferred Reporting Items for Systematic Reviews and Meta-Analyses (Moher et al., 2009) and was prospectively registered with PROSPERO (CRD42021228822; May 17, 2021).

### Search Strategy and Key Terms

We searched databases Medline Complete, APA PsycINFO, CINAHL Complete, and SocIndex via the EBSCOhost platform, EMBASE via Embase.com, and Maternity and Infant Care via Ovid for articles containing four concepts: fathers, intervention, IPV, and pregnancy/early childhood. We included relevant MeSH terms for Medline and similar subject headings in other databases. The search was conducted in May 2021. Search terms and strategies are in Supplementary Table 1. Relevant reviews on similar topics and included manuscripts were hand searched for additional relevant papers.

Titles and abstracts, and subsequently full-text articles, were screened in Covidence (Veritas Health Innovation, 2023) by two reviewers. Inter-rater reliability was acceptable: 97.52% for title and abstract screening and 81.20% for full text. Conflicts were settled by a third reviewer.

### Study Inclusion Criteria

The review was restricted to literature published in English in peer-reviewed journals, reporting on original quantitative research. There were no restrictions on publication date. Inclusion and exclusion criteria were guided by the following PICO framework: *Population* included men whose partners were expecting a baby or with at least one dependent child living at home. *Intervention* targeted fathers or parents. For interventions including mothers and fathers (“parents”), at least 50% of participants had to be fathers. *Comparator* comprised either a control group (i.e., group not receiving the intervention) or pre-intervention scores. *Outcomes* were any measure of prevalence or severity of IPV (e.g., violence, conflict, and abuse) perpetrated by adult men toward female intimate partners. IPV could be reported by the perpetrator, victim, or by a third party (e.g., official records). The first author contacted the authors of any articles in which it was necessary to clarify whether studies met these criteria.

### Quality Assessment

Methodological and reporting quality of the included manuscripts was assessed using the Quality Assessment with Diverse Studies (QuADS) tool (Harrison et al., 2021). Thirteen criteria are each rated on Likert scales from 0 to 3; total scores range from 0 to 39. Quality assessment was carried out by the first author; five manuscripts were simultaneously assessed by other members of the review team; inter-rater correlation was .62, which is similar to the .66 reported by the authors of the QuADS.

### Data Extraction

Data extracted included: study setting, aims, design, sampling pool and recruitment method, sample size and characteristics, number of participants, response fraction, type of participants (e.g., fathers, mothers, and fathers), measure of IPV, timing of assessments, intervention details (including theoretical or conceptual framework, content, format, and duration), and statistical results (e.g., unadjusted and adjusted effects or relevant data to calculate those scores). Where reported data were unclear, the first author attempted to contact the corresponding authors for clarification.

### Data Synthesis and Analysis

Study characteristics were summarized according to country, sample sizes, sampling methods, settings, intervention participants, follow-up assessment times, types of IPV assessed, sources of data, and assessment of IPV. We also noted whether studies were designed for primary prevention of IPV, primary prevention but including only participants known to be at high risk of perpetuating (men) or experiencing (women) IPV, or secondary prevention. Studies were categorized into two main groups according to design: first, repeated measures studies (no control group reported) and second, trials reporting data from both intervention and control groups. Studies in the latter group were further categorized into those which did not report any significant changes, and those reporting significant changes in the intervention versus the control group regarding at least some aspects of IPV. Findings were further reported according to timing between the interventions and the follow-up assessments as well as whether or not statistical analysis included adjustment for relevant covariates. Meta-analysis was not possible owing to extremely limited overlap in study designs, types of IPV assessed, statistical outcomes reported, and outcome assessment time-frames.

## Results

**Figure 1.**
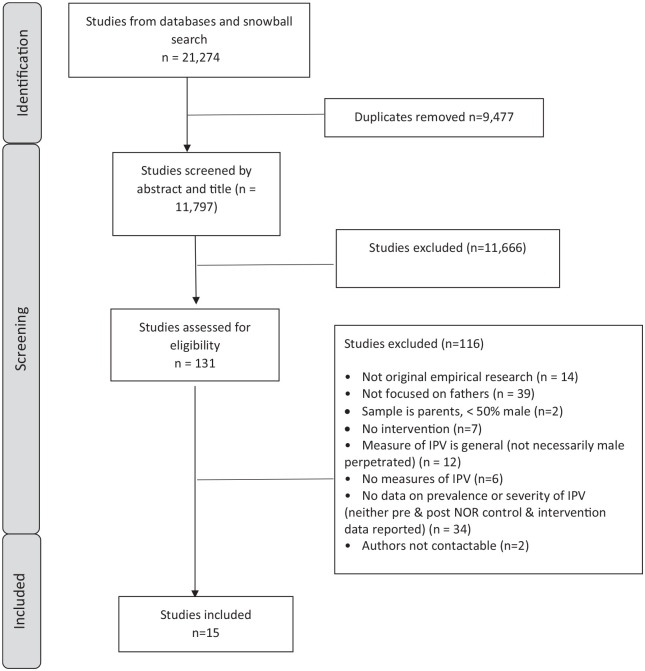
Preferred Reporting Items for Systematic Reviews and Meta-Analyses flow chart showing screening and identification of eligible papers.

Fifteen papers were eligible for inclusion (see Figure 1).

### Quality Assessment

Included studies were of poor-to-moderate quality (Supplementary Table 2). Total QuADS scores ranged from 13 to 27 and the median score was 22. Criteria that were satisfactorily addressed in the majority of studies included data collection tools assessed as appropriate to meet aims (*N* *=* 13 studies were coded as 2 or 3 on a scale of 0 (lowest) to 3 (highest)) and strengths and limitations being critically discussed (*N* *=* 12). However, several criteria were not consistently addressed across the studies: Aims and data collection procedures were clearly described in only seven studies each, theoretical or conceptual underpinnings for the intervention were provided in only four studies and clear sample size justification in only three. Only one study met the criterion of providing evidence of inclusion of research stakeholders (for example, fathers, families, and practitioners) in informing the intervention or study design.

### Study Characteristics

Study characteristics are summarized in Supplementary Table 3. Seven studies took place in the United States, two each in Rwanda, New Zealand, and Iran, and one each in Uganda and United Kingdom. Data from at least 10,816 participants were included; sample sizes ranged from 18 to 3,609; the median sample size was 368. Sampling methods included convenience (*N* *=* 9), purposive or referred (*N* *=* 4), systematic (*N* *=* 2), and snowball (*N* *=* 1); some studies used more than one sampling method. Settings included community health (*N* *=* 11) and specialized (*N* *=* 4) settings such as court-referred.

Of the 15 papers, three studies reported on interventions focused on fathers only, while the remaining studies targeted fathers and mothers (*N* *=* 12), of which one also included other caregivers of the infant.

Follow-up assessments were conducted immediately (*N* *=* 2), less than 12 months (*N* *=* 6), 12 to 24 months (*N* *=* 3), or longer, up to 9 years, after completion of the intervention (*N* *=* 4). Median follow-up duration was 7 months.

Types of violence assessed included physical (*N* *=* 10), psychological (*N* *=* 8), sexual (*N* *=* 4), economic/financial (*N* *=* 3), emotional (*N* *=* 2) and verbal (*N* *=* 2), and scores based on combinations of these (*N* *=* 8). Two studies indicated “severe” levels of IPV as an outcome variable.

In three of the studies, IPV data were reported by fathers only, while six studies reported data from mothers only. Other studies reported on data collected from both mothers and fathers (*N* *=* 2), or a combination of scores from both was used (*N* *=* 4).

In the studies that reported fathers’ age, the mean age ranged from 23 to 35.9. Two studies reported on interventions during pregnancy (Babaheidarian et al., 2021; Setodeh et al., 2019), two reported on interventions for parents who were *either* expecting a baby or had children living at home with them (Doyle et al., 2018; Rhoades, 2015) and the remaining studies targeted parents with children living at home (Table 1).

The most commonly used measure of violence reported was the Conflict Tactics Scale (*N* *=* 8) (Strauss et al., 1996), including original, revised, and study-specific shortened versions (Garcia-Moreno et al., 2006). See Table 1 for other measures reported.

**Table 1.** Overview of Studies Reporting on Father-Focused Interventions to Prevent or Reduce IPV.

Author(s) (Year); Country	Study Design; Prevention Type	Type of Participants; *N*; Child Age^a^	Intervention Name; Duration; Framework	IPV Measure; Reported By	IPV Post-Exposure Assessment Time Point; Results
Ashburn et al. (2017); Uganda	CT; Primary	Fathers; 500; 3 y (on average).	Responsible, Engaged, and Loving Fathers Initiative (REAL); 6 m, further details NR; Social cognitive theory.	CTS; fathers.	4 m and 8–12 m; Significantly lower psychological and verbal IPV, but not physical IPV, in the intervention group compared to the control group at follow-ups.
Babaheidarian et al. (2021); Iran	RCT; Secondary	Fathers (50%) and other family members; 90; pregnancy.	Greeting, Ask, Tell, Help, Explain, and Refer (GATHER); 3 × 45 min; NR.	Iran-specific instrument; mothers.	4 w; Significantly lower psychological, financial, physical, sexual, and social IPV, but not emotional IPV, in the intervention group compared to the control group at follow-up.
Doyle et al. (2018); Rwanda	RCT; Primary	Fathers (50%) and mothers; 2,398; (pregnancy and childhood, child age NR).	Bandebereho intervention; 15 w, further details NR; Sociological theories of gender and masculinities.	NR; mothers.	16 m; Significantly lower physical and sexual IPV in the intervention group compared to the control group at follow-up.
Fergusson et al. (2006); New Zealand	RCT; Primary (sample at risk)	Fathers (% NR) and mothers; 443 families; 0–3 m.	Early Start; up to 36 m, further details NR; Social learning model.	Revised CTS; mothers.	36 m; No significant differences in physical IPV between intervention and control groups.
Fergusson et al. (2013); New Zealand	RCT; Primary (sample at risk)	Fathers (% NR) and mothers; 443 families; 0–3 m.	Early Start; up to 36 m, further details NR; Social learning model.	Revised CTS; mothers and fathers.	5 y and 9 y (pooled effect); No significant differences in mother-reported IPV victimization scores or father-reported IPV perpetration scores between intervention and control groups.
Heyman et al. (2019); United States	RCT; Primary (sample at risk)	Fathers (50%) and mothers; 368 couples; 0–3 m.	Couple Care for Parents of Newborns (CCP); approximately 24 m, further details NR; NR.	Revised CTS; mothers.	8 m, 15 m, and 24 m; No significant differences in psychological or physical IPV between the intervention group and control group at any follow-up.
Heyman et al. (2020); United States	RM; Primary (sample at risk)	Fathers (% NR) and mothers; 443 couples; 0–3 m	CCP; 8 m; NR.	Revised CTS; aggregated mothers and fathers.	Immediate and 7 m; No significant differences in psychological or physical IPV from baseline to immediately post-intervention or 7 m follow-up.
Jensen et al. (2021); Rwanda	Cluster RCT; Primary	Fathers (70%) and mothers; 1,498 caregivers; child age NR.	Sugira Muryango intervention; 12 × 60 mins, weekly; UNICEF and WHO Child Development and Nurturing Care.	Rwanda NDHS Domestic Violence; mothers and fathers.	Immediate and 12 m; Significantly greater decrease in physical and sexual IPV from baseline to follow-ups in the intervention group compared to the control group, as reported by mothers but not fathers.
Kan et al. (2014); United States	RCT; Primary	Fathers (50%) and mothers; 169 couples; child age NR.	Family Foundations Program; NR; Developmental family systems.	Revised CTS (Adapted); aggregated mothers and fathers.	30 m; No significant differences in psychological IPV between intervention and control groups at follow-up.
McConnell et al. (2017); United Kingdom	RM; Secondary	Fathers (58%) and mothers; 271; 0–18 y (median 4y).	Caring Dads; 15 sessions; NR.	Controlling Behavior Inventory; mothers and fathers.	Immediate and 6 m; Significant reduction from pre-intervention to immediate post-intervention and/or 6 m post-intervention for father-reported “overall controlling behavior” and mother-reported total IPV, emotional abuse, violence, injury and denial minimization, but not intimidation, economic abuse, isolation, threat/coercion, sexual abuse, or using children.
Rhoades (2015); United States	RCT; Primary	Fathers (% NR) and mothers; 3,609; pregnancy or child age 0–18 y.	Within Our Reach; 28 h; NR.	Reports of IPV; mothers.	12 m and 30 m; Significantly lower psychological IPV, but not physical or severe physical IPV, at 12 m follow-up in intervention compared to control group. No significant differences in psychological, physical, or severe physical IPV at 30 m follow-up.
Setodeh et al. (2019); Iran	RCT; Primary	Fathers (50%) and mothers; 150 couples; pregnancy.	Attachment skills training; 4 w; Attachment.	Questionnaire previously used in Iran; mothers.	Immediate; Significantly lower social, sexual, and overall IPV in the intervention group compared to the control group immediately post-intervention. No significant differences in psychological, physical, or financial IPV.
Stover (2015); United States	RT, Secondary	Fathers; 18; 0–9 y (average 3 y).	Fathers for Change (F4C); 16 w; Fathering role is motivator for change.	Revised CTS (author correspondence); fathers.	Immediate and 3 m; Significantly lower IPV post-exposure compared to pre-exposure across both intervention groups (Fathers for Change (F4C) and individual drug counseling). No significant difference in reduction in physical aggression between the intervention groups.
Stover et al. (2019); United States	RT; Primary (sample at risk)	Fathers; 62; 0–15 y.	Fathers for Change (F4C); 12 w; Family systems, attachment, and Cognitive Behavioral Therapy.	CTS and TimeLine Follow-back-Spousal Violence Scale; fathers.	3 m; Significant reduction in psychological and physical IPV in the Fathers for Change (F4C) parenting intervention group. Significant reduction in total, psychological, and physical IPV in the alternative parenting intervention group. No significant differences in reduction in scores between F4C group and alternative parenting intervention group.
Stover et al. (2020); United States	RM; Secondary	Fathers (50%) and mothers; 204 couples; 0–18 y (average 9 y).	Fathers for Change (F4C); 18–24 w; Family systems, attachment and Cognitive Behavioral Therapy	Abusive Behavior Inventory total score; mothers.	Immediate; Significant reduction in scores from baseline to follow-up.

*Note.* CT = Controlled trial; CTS = Conflict tactics scale; h = hours; IPV = Intimate partner violence; m = months; min = minutes; NDHS = National Demographic Health Survey; NR = Not reported; Primary = primary prevention that is, targets all participants, not only those who have perpetrated or experienced IPV; Primary (at-risk sample) = primary prevention with specific at-risk sample that is, not limited to those who have perpetrated or experienced IPV, but participants are targeted because they have specific risk factors for IPV; RCT = Randomized controlled trial; RM = Repeated measures; RT = Randomized trial; Secondary = secondary prevention that is, targets those who have perpetrated or experienced IPV; y = years.

a Child age unless otherwise specified as pregnancy.

### Study Findings

Main findings are summarized in Tables 1 and 2; more detailed findings from each of the included studies are provided in Supplementary Table 3.

**Table 2.** Critical Findings from this Study.

1. There is scarce evidence (*N* *=* 15 papers) for the efficacy of father-inclusive interventions to prevent or reduce family violence during pregnancy and early parenthood. Existing evidence is mostly of poor-to-moderate quality.
2. Among studies that reported data from both intervention and control groups (*n* *=* 12), half did not report any significant differences in the prevalence or severity of IPV in the intervention versus the control groups. In the remainder, the intervention was associated with significantly greater reductions in some, but not all, types of IPV at follow-up time points from 0 to 24 months post-intervention.
3. Commonly, conceptual frameworks or theories underpinning interventions were not reported. Among the three controlled trials reporting in adjusted analyses a difference or change in any form of IPV at any time point, two were based on challenging gender norms.

*Note.* IPV = intimate partner violence.

**Repeated measures studies with no control group** (Table 1). Three papers reported on studies using repeated measures designs, in a single sample with no IPV data from a control group reported. Analyses were not adjusted for relevant demographic or reproductive variables in any of these studies. In the United Kingdom, McConnell et al. (2017) reported reductions sustained to 6 months post-intervention in emotional abuse, violence, denial minimization, injury, and total controlling behavior score, but not in intimidation, economic abuse, isolation, threat/coercion, or using children. In the U.S., Stover et al. (2020) reported reduced IPV among those who completed all components of the intervention, and for those who did not; mean scores on the Abusive Behavior Inventory were still in the “abusive” range for non-completers. The latter two studies (McConnell et al., 2017; Stover et al., 2020) both represent secondary prevention approaches in which participants were selected because of the existence of IPV in their relationships. In the U.S., Heyman et al. (2020) found no significant changes over time using a primary prevention approach among an at-risk sample of low-income, unmarried perinatal couples.

**Studies reporting data from both intervention and control groups**(Table 1). Twelve studies reported data from both intervention and control/other intervention groups; designs included randomized and non-randomized controlled trials and cluster randomized trials. Of these, five (in New Zealand and the United States) did not report any significant differences in prevalence or severity of IPV in the intervention versus the control group (Fergusson et al., 2013, (Fergusson, Grant, Horwood and Ridder, 2006; Heyman et al., 2019) or compared to other interventions (Stover, 2015; Stover et al., 2019). Target groups were families with risk factors for poor parenting and family outcomes (Fergusson et al., 2006, 2013), couples reporting verbal aggression in the past 6 months (Heyman et al., 2019), fathers being treated for substance use disorders (Stover et al., 2019) or men with histories of substance abuse and IPV (Stover, 2015). An additional paper reported no main effect for the intervention in an unselected sample in the U.S., but in stratified analysis, fathers with high preprogram aggression in the intervention group demonstrated significantly greater reduction in IPV than the control group (Kan & Feinberg, 2014).

Among the six studies reporting any significant results in at least some types of IPV between intervention and control groups, two studies reported simple, unadjusted bivariate differences between control and intervention groups at short follow-up points: In Iran, Setodeh et al. (2019) reported less social, sexual, and overall violence in the intervention compared to the control group immediately after the intervention, but no differences in physical, psychological, or financial violence. Also in Iran, 4 weeks after an intervention, Babaheidarian et al. (2021) found less sexual, physical, verbal, financial, social, and economic violence and lower total domestic violence scores, but not emotional violence, in the intervention group compared to the control group.

Four of the six studies reporting significant differences between intervention and control groups followed up with participants for longer periods. In a simple, unadjusted bivariate analysis between control and intervention groups at 12 months after an intervention in the U.S., Rhoades (2015) reported significantly less psychological IPV, but not physical IPV, in the intervention compared to the control group; however, these differences were no longer evident at 30 months post-intervention. The remaining three studies reported significant differences in statistical models adjusted for relevant covariates. In Uganda, Ashburn et al. (2017) reported a significantly greater reduction in psychological and verbal but not physical IPV by fathers in the intervention group compared to the control group at 4 and 8–12 months post-intervention. In Rwanda, Doyle et al. (2018) reported that at 21 months post-intervention, there was a significantly greater reduction in female-reported partner physical and sexual violence, compared to the control group. Also in Rwanda, Jensen et al. (2021) reported significantly lower incident risk ratios for female-reported (but not male-reported) physical and/or sexual violence, immediately after the intervention and at 12 months post-intervention.

Notably, five of the six studies reporting results from both control and intervention groups were designed for primary prevention of IPV. The study by Babaheidarian et al. (2021) was the only secondary prevention study among them.

**Theoretical underpinnings or conceptual frameworks for successful interventions.** Among the interventions reported to show significant change or difference in any form of IPV at any time point, two were based on challenging gender norms, focusing on human rights and/or gender equity (Ashburn et al., 2017; Doyle et al., 2018). “Father attachment,” “Relationship education” and “The World Health Organization’s Child Development and Nurturing care bundle’ were the basis for the interventions described by Setodeh et al. (2019), Rhoades (2015) and Jensen et al. (2021) respectively, but further details of the conceptual underpinning of their interventions were not reported. Theoretical frameworks in the remaining studies reporting significant effects were not clearly described or were absent (Babaheidarian et al., 2021; McConnell et al., 2017; Stover et al., 2020).

## Discussion

This review is an informative response to the call for rigorous evidence on effectiveness of interventions targeting men for the prevention or reduction of IPV (Heward-Belle et al., 2019), especially during the critical life transition to becoming a parent (D’Inverno et al., 2018; Humphreys et al., 2020; Stanley & Humphreys, 2017). Despite the higher prevalence of IPV during pregnancy (Román-Gálvez et al., 2021) and the early parenting period (Hasstedt & Rowan, 2016) than at other life stages, there are very few studies on interventions specifically targeting new or expectant fathers. This is concerning, as the transition to parenthood provides an opportunity to work with men who perpetrate IPV, at a time when they may be more motivated to change their behavior ((Australia’s National Research Organization for Women’s Safety (ANROWS) 2020a) Cooper et al., 2013; Labarre et al., 2016; Oliffe et al., 2022; Stanley et al., 2012). In addition, intervening during the early parenting period could have longer-term impacts on violence prevention by interrupting intergenerational cycles, which arise in part because men who perpetrate violence have themselves experienced violence in their childhood (Capaldi et al., 2012). The outcomes of this review align with other systematic reviews of general IPV interventions not focused on the perinatal period, which also assert that there are remarkably few studies investigating the effectiveness of interventions for men who perpetrate violence, and evidence is inconclusive owing to methodological challenges (Karakurt et al., 2019; Travers et al., 2021; Yakeley, 2022).

The highest quality evidence of successful interventions was reported in three controlled trials reporting adjusted odds, risk, or incidence ratios with 95% confidence intervals to show significant differences between the *reduction* in IPV in intervention and control groups, sustained to at least 12 months after the intervention in Uganda and Rwanda (Ashburn et al., 2017; Doyle et al., 2018; Jensen et al., 2021). Notably, these three trials represented primary prevention approaches, consistent with the overall evidence that primary prevention approaches can effectively prevent IPV in a more sustainable way (Alsina et al., 2023; Hardesty & Ogolsky, 2020) as they address broader, structural, and social risk factors rather than being primarily limited to individual- or couple-level risk factors (Beyer et al., 2013). Similarly, large-scale primary prevention approaches have reported significantly greater reductions in IPV among intervention than control participants in couple-focused interventions not limited to parents of young children in Rwanda (Dunkle et al., 2020) and India (Raj et al., 2016). Owing to their prevention focus, these interventions have potentially far-reaching benefits for women, children, men, and communities (World Health Organization (WHO) 2012) but require substantial, multi-sectoral investment to achieve broad social change, which also makes them more difficult to assess (World Health Organization (WHO) 2012. Secondary prevention approaches may be narrower in scope to assess but present an additional barrier in that demonstrating statistically significant changes in IPV prevalence or severity is difficult in perpetrator programs in which decisions about the existence or nature of “control” groups may represent an ethical dilemma. In addition, participants referred (e.g., by court) to secondary prevention programs may be reluctant to attend or persist with programs (Augusta-Scott & Dankwort, 2002), which also presents challenges in terms of recruiting and sustaining sufficient sample sizes.

All of these effective primary prevention interventions in perinatal populations (Ashburn et al., 2017; Doyle et al., 2018; Jensen et al., 2021) involved at least some work with couples to address child well-being and/or a focus on transforming gender relations within the couple, which is reported to be a common element in interventions to prevent IPV (Kerr-Wilson et al., 2020). While group and community work, found to be effective in IPV evaluations in general (Jewkes et al., 2020; Leight et al., 2023), featured in some of these trials, they did not form part of all of these interventions. In addition, group and community work featured in other included studies that did not report statistically significantly greater reduction in IPV in intervention than in control groups. The lack of statistically significant effects may be because of small sample sizes (Stover, 2015; Stover et al., 2019), poor intervention completion rates resulting in participants receiving a diluted or partial intervention (Heyman et al., 2019), ambitious program aims that incorporate broad child health and development as well as parenting outcomes (Fergusson et al., 2006, 2013), or not directly targeting violence-specific cognitions and behaviors (Kan & Feinberg, 2014).

Although a recent meta-analysis found no significant differences in the effectiveness of IPV interventions between low- or middle-income countries compared to high-income countries (Alsina et al., 2023), we note that the most robust statistical evidence for effective IPV interventions including fathers came from low- and middle-income countries in which traditionally, men are less involved in family life (Barker et al., 2021). In Rwanda, the Ministry of Health has prioritized engaging men in health care during the perinatal period and in the postnatal care of their children, while maintaining an ongoing focus on maternal rights and well-being (Barker et al., 2021). Both controlled trials conducted in Rwanda reported effective interventions. There are a number of potential explanations for the reported success of programs in such settings, including theories of change, which focus on transforming traditional, rigid gender norms that exist at the societal level (Gibbs, Dunkle, Mhlongo et al., 2020; Jewkes et al., 2021). In addition, the higher prevalence of IPV in the community under investigation makes it more likely that change will be demonstrated when the rate and severity of violence are higher than in other regions at baseline, as is the case for these lower- and middle- income countries (Mojahed et al., 2021; Román-Gálvez et al., 2021; Sardinha et al., 2022; WHO, 2021).

Consistent with findings of reviews of interventions targeting men across the lifespan (Maheu-Giroux et al., 2022), our review of IPV interventions during pregnancy and early parenting found substantial variation in the quality of methods, measures, and reporting (Babcock et al., 2016; Babcock, Green, Webb et al., 2004; Brown & James, 2014; Koehler et al., 2013). For example, some studies provided data separately for program “completers” or “compliers” versus “non-completers” or “non-compliers” (Heyman et al., 2019; Stover et al., 2019); these data are not readily interpreted when aiming to assess the efficacy of intervention. In addition, effect size data, or data that enables the calculation of effect sizes, were often not provided in published manuscripts; these data were requested from the authors, but not always provided. The poor-quality reporting of IPV outcome data in some studies included in this review makes comparisons of effectiveness and the synthesis of recommendations for the development of further programs very difficult. While more “nuanced” reporting of IPV measures than binary (experienced or perpetrated versus none) is recommended in IPV program evaluations (Chatterji et al., 2023), only two studies (Heyman et al., 2020; Rhoades, 2015) in the current review reported “severe” violence of any sort as an outcome. An additional four studies reported continuous scores on an IPV assessment tool (Fergusson et al., 2013; McConnell et al., 2017; Stover, 2015; Stover et al., 2020). All the remaining studies reported binary outcome measures; this may limit evaluation of whether a program is perceived to be effective (Chatterji et al., 2023).

While we recognize the importance of services and support for women and children living with violence, we expected to find more studies reporting on interventions specifically targeting or including fathers in the family’s transition to parenthood. A 2016 review of strategies for the prevention of IPV during the childbearing years (Sinnott & Artz, 2016) reported a “disproportionate emphasis on making females responsible for not becoming victims of IPV. . .Also noted is a striking lack of research on the prevention of IPV perpetration in males, particularly fathers” (p. 324). This gap has still not been addressed. It is difficult to understand how real reductions in rates of IPV can be achieved without working with men to effect behavior change among those who perpetrate IPV, although program effectiveness is of course ultimately dependent upon the commitment of participants to changing their violent behaviors (Augusta-Scott & Dankwort, 2002; Bowen & Gilchrist, 2006; Brown & James, 2014; McGinn et al., 2020). Two of the three controlled trials reporting significant, adjusted differences in IPV sustained to at least 12 months post-intervention, were based on social cognition and gender role transformation (Ashburn et al., 2017; Doyle et al., 2018). Subsequently, one of these programs, a gender-transformative couples’ intervention (Doyle et al., 2018) has reported sustained effects at 6-year follow-up; women in the intervention group reported significantly less physical, sexual, economic, and emotional IPV at 6 years following the intervention (Doyle et al., 2023). It is, therefore, evident that helping men to question learned attitudes and historical role models and to change their cognitions about their roles and the roles of their partners may be an effective way to reduce IPV (Gibbs, Dunkle, Mhlongo et al., 2020; Jewkes et al., 2021). However, men in any culture or society are likely to carry a wide range of beliefs and experiences, and interventions can have differential impacts for different individuals; for example, in studies not limited to the perinatal period, Gibbs et al. (2020) identified that some men with depression symptoms, living away from their partner, with recent employment and lower perceived acceptability of IPV, responded better to IPV interventions than other men (Gibbs, Dunkle, Mhlongo et al., 2020). Programs have also been reported to be more effective in reducing IPV among men with higher (Kan & Feinberg, 2014) or lower (Christofides et al., 2020) pre-intervention levels of violence; these contradictory results could be due to diverse program characteristics, sampling approaches, and settings. It will, therefore, be important to consider targeting interventions and engaging men according to individuals, their personal histories, and other characteristics that may bolster or inhibit intervention effects (Florsheim et al., 2011; Gibbs, Dunkle, Mhlongo et al., 2020; Heyman et al., 2019).

Other important outcomes related to IPV interventions, not the focus of this review, were reported. For example, some reported interventions increased men’s attendance and accompaniment at antenatal care (Doyle et al., 2018), improved emotion regulation and reflective functioning as well as reduced anger and hostility among men (Stover et al., 2020), reduced child physical punishment (Doyle et al., 2018; Fergusson et al., 2013; Stern et al., 2022), and increased men’s participation in childcare and household tasks (Doyle et al., 2018). These outcomes are important in their own right because they are likely to facilitate enhanced health outcomes for women, men, and children; however, they may also be important mechanisms for change in men’s behavior and their relationships with their partners and children (Flynn, 2012; World Health Organization (WHO) 2012). The WHO (World Health Organization (WHO) 2019) refers to “interim indicators,” which contribute long-term toward reductions in the prevalence of violence against women, including gender equitable attitudes and norms; the social acceptance of IPV among women and men has been reported to be significantly reduced by interventions in studies not limited to pregnancy or the early parenting period (Abramsky et al., 2014; Dunkle et al., 2020; Raj et al., 2016). Longer-term follow-up studies may be needed to assess these and any subsequent changes in IPV outcomes. Cognitions, attitudes, and behaviors may all need to change for an intervention to be “successful” (Abramsky et al., 2014; Fernández-Montalvo et al., 2015, 2020). It is encouraging that prevention programs are targeting boys and younger men (Corboz et al., 2019; Jewkes et al., 2011) to challenge inequitable gender norms and build healthy relationships before young men become involved in intimate relationships.

### Limitations

In this study, we aimed to focus on peer-reviewed reports and did not include gray literature. Gray literature may include studies with null or negative results, and thus provide a more complete picture of available evidence (Paez, 2017). Although many of the included studies scored poorly to moderately on quality assessment, these were still included, owing to the small number of papers eligible for inclusion in this review.

Although other studies reported interventions to reduce or prevent IPV among new parents, these were excluded as they provided only household-level data (Barnhart et al., 2020; Feinberg et al., 2016; Florsheim et al., 2011), and it was not possible to extract data about IPV carried out by men.

A limitation of the literature that was available to be reviewed is that most estimates of IPV in the included study are based on women’s or men’s self-reported experiences of being subjected to, or perpetrating, IPV. Given the sensitive nature of the issue and the tendency of women and men to underreport experiences of IPV, the true prevalence of IPV is likely to be higher.

### Implications

Working with men’s beliefs about gender and parenting roles in their societal and cultural context may increase the relevance of interventions to men’s specific contexts (Ashburn et al., 2017; Doyle et al., 2018; Dunkle et al., 2020; Raj et al., 2016). Including a range of stakeholders such as fathers, families, health professionals, and health service leaders in the design of interventions and research evaluating their effectiveness is important, as they may provide more insight into what might or might not work or is appropriate for the target population. Our quality assessment identified only one study that did this (Jensen et al., 2021); this intervention was effective in reducing IPV.

More evidence is needed on interventions during pregnancy, given that worldwide, one in four women experience IPV during pregnancy (Román-Gálvez et al., 2021). We found only four studies that targeted men during pregnancy (Babaheidarian et al., 2021; Doyle et al., 2018; Rhoades, 2015; Setodeh et al., 2019). Pregnancy provides a unique opportunity to engage with men at a time when most couples are in regular contact with health care providers (Johnston et al., 2015; Van Parys et al., 2014) and potentially a time when men may be open to behavior change (Oliffe et al., 2022).

Studies assessing the effectiveness of interventions to prevent or reduce IPV during the transition to parenthood should adhere to better standards of reporting. In line with recommendations that all IPV prevention programs should specify an underlying theory of change (WHO, 2019), studies should report on the theoretical underpinnings of the intervention, along with detailed methodology and clarity in the reporting of key factors such as implementation, participation, and evaluation. Specifically, studies should report evidence of sufficient sample power, use of evidence-based measures of IPV, and adequate and transparent analysis and reporting of data, including effect sizes in statistical analysis adjusted for relevant covariates.

In summary, despite the potentially disastrous impact of IPV against women on health and well-being of men (Yu et al., 2019), women, and children (Finnbogadóttir et al., 2020; Pastor-Moreno et al., 2020), we identified only 15 studies reporting on interventions targeting fathers to prevent or reduce IPV. Of these, twelve reported designs including a control / alternative intervention group, of which six reported significant differences between intervention and control groups immediately after the intervention or at subsequent follow-up assessments. Women are at elevated risk of IPV in this period, and it is also a period when many couples are in regular contact with health care providers and open to influence. Worldwide, inclusion of fathers in maternal and child health and early parenting interventions is recommended, for the benefit of women, men, children, and family functioning (WHO, 2015), but it is essential that interventions that involve fathers routinely are designed and monitored to ensure that they do not cause harmful effects on couple relationships, reinforce unequal gender norms and norms related to caring for infants, or compromise women’s autonomy (Comrie-Thomson et al., 2021; Tokhi et al., 2018; WHO, 2015). There is an urgent need to develop effective intervention strategies for men who are likely to perpetrate violence in intimate relationships across the transition to parenthood and to assess and report on the effectiveness of these interventions in a robust and transparent way (Table 3).

**Table 3.** Implications for Practice, Policy, and Research.

1. As the transition to parenthood is an excellent opportunity to intervene with men who perpetrate or are likely to perpetrate IPV, more interventions should be developed that target or include fathers.
2. Interventions should be based on conceptual frameworks which include challenging perceived, “traditional” gender norms.
3. High-quality studies and reporting of outcomes are needed in both resource-constrained and high-income settings.

*Note.* IPV = intimate partner violence.

## Supplemental Material

sj-docx-1-tva-10.1177_15248380241277270 – Supplemental material for Effectiveness of Father-Focused Interventions to Prevent or Reduce Intimate Partner Violence During Pregnancy and Early Parenthood: A Systematic Review Supplemental material, sj-docx-1-tva-10.1177_15248380241277270 for Effectiveness of Father-Focused Interventions to Prevent or Reduce Intimate Partner Violence During Pregnancy and Early Parenthood: A Systematic Review by Karen Wynter, Lauren M. Francis, Ashlee Borgkvist, Barnaby Dixson, Levita D’Souza, Elisabeth Duursma, Chris May, Louisa Sher and Jennifer St George in Trauma, Violence, & Abuse

sj-docx-2-tva-10.1177_15248380241277270 – Supplemental material for Effectiveness of Father-Focused Interventions to Prevent or Reduce Intimate Partner Violence During Pregnancy and Early Parenthood: A Systematic Review Supplemental material, sj-docx-2-tva-10.1177_15248380241277270 for Effectiveness of Father-Focused Interventions to Prevent or Reduce Intimate Partner Violence During Pregnancy and Early Parenthood: A Systematic Review by Karen Wynter, Lauren M. Francis, Ashlee Borgkvist, Barnaby Dixson, Levita D’Souza, Elisabeth Duursma, Chris May, Louisa Sher and Jennifer St George in Trauma, Violence, & Abuse

sj-docx-3-tva-10.1177_15248380241277270 – Supplemental material for Effectiveness of Father-Focused Interventions to Prevent or Reduce Intimate Partner Violence During Pregnancy and Early Parenthood: A Systematic Review Supplemental material, sj-docx-3-tva-10.1177_15248380241277270 for Effectiveness of Father-Focused Interventions to Prevent or Reduce Intimate Partner Violence During Pregnancy and Early Parenthood: A Systematic Review by Karen Wynter, Lauren M. Francis, Ashlee Borgkvist, Barnaby Dixson, Levita D’Souza, Elisabeth Duursma, Chris May, Louisa Sher and Jennifer St George in Trauma, Violence, & Abuse
