# Supplementary material for: Effectiveness of Father-Focused Interventions to Prevent or Reduce Intimate Partner Violence During Pregnancy and Early Parenthood: A Systematic Review
Source: Trauma Violence Abuse. 2024 Sep 20;26(1):167–82. doi: 10.1177/15248380241277270 (PMC11558938; doi:10.1177/15248380241277270)
Supplement: sj-docx-1-tva-10.1177_15248380241277270 – Supplemental material for Effectiveness of Father-Focused Interventions to Prevent or Reduce Intimate Partner Violence During Pregnancy and Early Parenthood: A Systematic Review [file sj-docx-1-tva-10.1177_15248380241277270.docx]

**Supplementary Table 1**

*Example search strategy (PsycINFO)*

Father/parent/couple AND intervention AND Violence AND Postnatal/pregnancy

| **#** | **Searches** | **results** |
| --- | --- | --- |
|  | S23 AND S42 AND S74 AND S102  [Father AND violence AND intervention AND postpartum] | 5,938 |
| S102 | S75-101  [first 1000? Days] | 855,570 |
| S101 | DE "Expectant Parents" | 198 |
| S100 | DE "Pregnancy" | 46,756 |
| S99 | DE "Perinatal Period" | 3,071 |
| S98 | DE "Postnatal Period" | 5,031 |
| S97 | DE "Birth" | 7,827 |
| S96 | TI “new parent*” OR AB “new parent*” | 773 |
| S95 | TI “new born*” OR AB “new born*” | 389 |
| S94 | TI newborn* OR AB newborn* | 11,169 |
| S93 | TI “transition to parent*” OR AB “transition to parent*” | 1,278 |
| S92 | TI babies OR AB babies | 16,896 |
| S91 | TI baby* or AB baby* | 12,891 |
| S90 | TI toddler* or AB toddler* | 9,581 |
| S89 | TI child* or AB child* | 706,873 |
| S88 | TI infan* or AB infan* | 93,700 |
| S87 | TI born or AB born | 32,927 |
| S86 | TI birth* OR AB birth* | 62,266 |
| S85 | TI “post-partum” OR AB “post-partum” | 1,272 |
| S84 | TI postpartum OR AB postpartum | 12,123 |
| S83 | TI “post-natal” OR AB “post-natal” | 1,126 |
| S82 | TI postnatal OR AB postnatal | 20,273 |
| S81 | TI “pre-natal” OR AB “pre-natal” | 237 |
| S80 | TI prenatal OR AB prenatal | 18,857 |
| S79 | TI “peri-natal” OR AB “peri-natal” | 66 |
| S78 | TI perinatal OR AB perinatal | 10,699 |
| S77 | TI “ante-natal” OR AB “ante-natal” | 59 |
| S76 | TI antenatal OR AB antenatal | 3,658 |
| S75 | TI pregnan* OR AB pregnan* | 47,825 |
| S74 | S43-72  [intervention] | 2,135,856 |
| S73 | DE "Training" | 19,571 |
| S72 | DE "Psychoeducation" | 4,772 |
| S71 | DE "Educational Therapy" | 202 |
| S70 | DE "Client Education" | 4,165 |
| S69 | DE "Coeducation" | 300 |
| S68 | DE "Consumer Education” | 212 |
| S67 | DE "Educational Programs” | 27,651 |
| S66 | DE "Family Life Education" | 317 |
| S65 | DE "Health Literacy" | 3,731 |
| S64 | DE "Mental Health Literacy" | 468 |
| S63 | DE "Intervention" | 68,872 |
| S62 | DE "Early Intervention" | 11,140 |
| S61 | DE "Family Intervention" | 3,388 |
| S60 | DE "Group Intervention" | 2,882 |
| S59 | DE "Treatment" | 76,335 |
| S58 | DE "Counseling" | 29,927 |
| S57 | TI treatment OR AB treatment | 633,227 |
| S56 | TI therap* OR AB therap* | 396,440 |
| S55 | TI “psycho-educat*” OR AB “psycho-educat*” | 2,230 |
| S54 | TI psychoeducat* OR AB psychoeducat* | 9,473 |
| S53 | TI seminar* OR AB seminar* | 7,900 |
| S52 | TI workshop* OR AB workshop* | 17,333 |
| S51 | TI literac* OR AB literac* | 31,824 |
| S50 | TI educat* OR AB educat* | 496,391 |
| S49 | TI counsel* OR AB counsel* | 107,396 |
| S48 | TI train* OR AB train* | 327,216 |
| S47 | TI pilot* or AB pilot* | 50,951 |
| S46 | TI trial* or AB trial* | 187,810 |
| S45 | TI strateg* or AB strateg* | 347,172 |
| S44 | TI program* or AB program* | 404,321 |
| S43 | TI interven* OR AB interven* | 411,748 |
| S42 | S24-41  [Domestic violence] | 74,434 |
| S41 | DE "Intimate Partner Violence” | 12,361 |
| S40 | DE "Domestic Violence" | 13,180 |
| S39 | TI “gender* based violen*” OR AB “gender* based violen*” | 786 |
| S38 | TI “partner monitor*” OR AB “partner monitor*” | 13 |
| S37 | TI "DV" OR AB "DV" | 994 |
| S36 | TI "IPV" OR AB "IPV" | 5,558 |
| S35 | TI batter* OR AB batter* | 42,884 |
| S34 | TI “parent* conflict*” OR AB “parent* conflict*” | 1,078 |
| S33 | TI “family conflict*” OR AB “family conflict*” | 4,901 |
| S32 | TI “spous* abuse” OR AB “spous* abuse” | 676 |
| S31 | TI “partner abuse” OR AB “partner abuse” | 865 |
| S30 | TI "domestic abuse" OR AB "domestic abuse" | 679 |
| S29 | TI “spous* violen*” OR AB “spous* violen*” | 237 |
| S28 | TI “marital violen*” OR AB “marital violen*” | 477 |
| S27 | TI "interpartner violen*" OR AB "interpartner violen*" | 15 |
| S26 | TI "partner violen*" OR AB "partner violen*" | 9,548 |
| S25 | TI "domestic violen*" OR AB "domestic violen*" | 9,847 |
| S24 | TI “family violen*” or AB “family violen*” | 3,079 |
| S23 | S1 OR S2 OR S3 OR S4 OR S5 OR S6 OR S7 OR S8 OR S9 OR S10 OR S11 OR S12 OR S13 OR S14 OR S15 OR S16 OR S17 OR S18 OR S19 OR S20 OR S21 OR S22  [Father OR Parent OR Couple] | 473,986 |
| S22 | DE "Spouses" | 14,654 |
| S21 | DE "Marriage" | 15,928 |
| S20 | DE "Couples" | 14,407 |
| S19 | DE "Husbands" | 2,283 |
| S18 | DE "Fathers" | 12,242 |
| S17 | DE "Expectant Fathers" | 199 |
| S16 | DE "Significant Others" | 1,386 |
| S15 | DE "Parents" | 55,302 |
| S14 | DE "Parenting" | 18,927 |
| S13 | DE "Coparenting" | 621 |
| S12 | TI “co-parent*” OR AB “co-parent*” | 660 |
| S11 | TI coparent* OR AB coparent* | 1,037 |
| S10 | TI “significant other*” OR AB “significant other*” | 6,470 |
| S9 | TI couple* OR AB couple* | 62,136 |
| S8 | TI spous* or AB spous* | 21,914 |
| S7 | TI Partner* OR AB partner* | 107,713 |
| S6 | TI parent* OR AB parent* | 276,547 |
| S5 | TI patern* OR AB patern* | 14,009 |
| S4 | TI husband* OR AB husband* | 13,460 |
| S3 | TI father* OR AB father* | 47,844 |
| S2 | TI dads OR AB dads | 934 |
| S1 | TI dad OR AB dad | 934 |
