# Supplementary material for: Effectiveness of Father-Focused Interventions to Prevent or Reduce Intimate Partner Violence During Pregnancy and Early Parenthood: A Systematic Review
Source: Trauma Violence Abuse. 2024 Sep 20;26(1):167–82. doi: 10.1177/15248380241277270 (PMC11558938; doi:10.1177/15248380241277270)
Supplement: sj-docx-2-tva-10.1177_15248380241277270 – Supplemental material for Effectiveness of Father-Focused Interventions to Prevent or Reduce Intimate Partner Violence During Pregnancy and Early Parenthood: A Systematic Review [file sj-docx-2-tva-10.1177_15248380241277270.docx]

**Supplementary Table 2**

*Quality Assessment Using the QuADS Tool*

|  | 1. Theoretical or conceptual underpinning to the intervention | 2. Explicit and specific statement of research aim/s | 3. Clear description of research setting and target population | 4. Study design is appropriate to address the stated research aim/s | 5. Intervention comprehensively described | 6. Sample size calculation (to address the research aim/s) and key sample characteristics adequately described | 7. Appropriate rationale presented for choice of data collection tool/s | 8. Format and content of data collection tool for IPV outcome/s are appropriate to address the stated research aim/s | 9. Data collection procedure adequately described | 10. Recruitment data provided (response rate and attrition) | 11. Method of analysis is appropriate to answer the research aim/s | 12. Evidence provided that the research stakeholders (fathers, families, services, practitioners) have been considered in intervention and/or research design or conduct | 13. Strengths and limitations critically discussed | Total score (Range 0 - 39) |
| --- | --- | --- | --- | --- | --- | --- | --- | --- | --- | --- | --- | --- | --- | --- |
| Ashburn et al. 2017 | 3 | 1 | 3 | 2 | 3 | 1 | 3 | 3 | 1 | 1 | 0 | 1 | 3 | 25 |
| Babahedarian et al. 2021 | 0 | 1 | 1 | 1 | 1 | 1 | 3 | 2 | 1 | 1 | 1 | 0 | 1 | 14 |
| Doyle et al. 2018 | 1 | 1 | 2 | 2 | 2 | 3 | 2 | 3 | 2 | 3 | 3 | 0 | 3 | 27 |
| Fergusson et al. 2013 | 1 | 3 | 2 | 3 | 1 | 1 | 0 | 1 | 1 | 3 | 2 | 0 | 1 | 19 |
| Fergusson et al. 2006 | 1 | 2 | 2 | 3 | 1 | 1 | 0 | 2 | 3 | 3 | 2 | 0 | 2 | 22 |
| Heyman et al. 2020 | 1 | 1 | 2 | 1 | 3 | 0 | 2 | 3 | 1 | 3 | 1 | 0 | 3 | 21 |
| Heyman et al. 2019 | 1 | 1 | 2 | 2 | 3 | 1 | 2 | 3 | 1 | 3 | 2 | 1 | 3 | 25 |
| Jensen et al. 2021 | 2 | 2 | 1 | 3 | 2 | 2 | 2 | 1 | 2 | 2 | 2 | 2 | 2 | 25 |
| Kan & Feinberg 2014 | 2 | 1 | 1 | 3 | 2 | 0 | 1 | 3 | 2 | 1 | 3 | 0 | 3 | 22 |
| McConnell et al. 2017 | 1 | 2 | 1 | 1 | 1 | 0 | 2 | 2 | 1 | 2 | 1 | 0 | 2 | 16 |
| Rhoades 2015 | 0 | 1 | 2 | 2 | 2 | 1 | 0 | 3 | 2 | 2 | 1 | 0 | 3 | 19 |
| Setodah et al. 2019 | 0 | 2 | 2 | 1 | 2 | 1 | 0 | 3 | 1 | 0 | 1 | 0 | 0 | 13 |
| Stover et al. 2015 | 1 | 1 | 2 | 2 | 2 | 1 | 0 | 2 | 2 | 3 | 1 | 0 | 2 | 19 |
| Stover et al. 2019 | 1 | 2 | 3 | 3 | 3 | 2 | 3 | 2 | 2 | 0 | 2 | 0 | 2 | 25 |
| Stover et al. 2020 | 2 | 3 | 1 | 1 | 2 | 1 | 1 | 3 | 1 | 3 | 2 | 0 | 2 | 22 |

NA – not applicable, secondary analysis (score adjusted)

Scores of 0 and 1 are shaded grey for ease of interpretation
