# Supplementary material for: Effectiveness of Father-Focused Interventions to Prevent or Reduce Intimate Partner Violence During Pregnancy and Early Parenthood: A Systematic Review
Source: Trauma Violence Abuse. 2024 Sep 20;26(1):167–82. doi: 10.1177/15248380241277270 (PMC11558938; doi:10.1177/15248380241277270)
Supplement: sj-docx-3-tva-10.1177_15248380241277270 – Supplemental material for Effectiveness of Father-Focused Interventions to Prevent or Reduce Intimate Partner Violence During Pregnancy and Early Parenthood: A Systematic Review [file sj-docx-3-tva-10.1177_15248380241277270.docx]

**Supplementary Table 3**

*Detailed Results: Father-Inclusive Interventions to Prevent or Reduce IPV*

| **Authors (year)**  **Country** | **Study design** | **Result summary. Outcome time point (reported by): Effect size, significance, analytic *n*** |
| --- | --- | --- |
| Ashburn et al. (2017)  Uganda | CT | Significantly lower psychological and verbal IPV, but not physical IPV, in the intervention group compared to the control group at follow-ups.  4m (father report):  Any IPV: OR^†^ = 0.48, CI (0.31, 0.76), *p* < 0.01, *n* = 435; Physical IPV: OR^†^ = 0.71, CI (0.46, 1.11), NS, *n* = 435; Psychological IPV: OR^†^ = 0.55, CI (0.35, 0.86), *p* < 0.01, *n* = 435; Verbal IPV: OR^†^ = 0.51, CI (0.33, 0.79), *p* < 0.01, *n* = 435.  8-12m (father report):  Any IPV: OR^†^ = 0.48, CI (0.31, 0.77), *p* < 0.01, *n* = 399; Physical IPV: OR^†^ = 0.76, CI (0.38, 1.35), NS, *n* = 399; Psychological IPV: OR^†^ = 0.42, CI (0.24, 0.74), *p* < 0.001, *n* = 399; Verbal IPV: OR^†^ = 0.56, CI (0.35, 0.90), *p* < 0.01, *n* = 399. |
| Babaheidarian et al. (2021)  Iran | RCT | Significantly lower psychological, financial, physical, sexual, social IPV, but not emotional IPV, in the intervention group compared to the control group at follow-up.  4w (mother report):  Emotional IPV: *t* = NR, *p* = 0.92, *n* = 90; Psychological, verbal, financial, physical, sexual, and overall IPV: *t* = NR, *p* < 0.001, *n* = 90; Social IPV: *t* = NR, *p* = 0.04, *n* = 90. |
| Doyle et al. (2018)  Rwanda | RCT | Significantly lower physical and sexual IPV in the intervention group compared to the control group at follow-up.  16m (mother report):  Physical IPV: OR^†^ = 0.37, CI (0.28, 0.49), *p* < 0.001, *n* = 1162; Sexual IPV: OR^†^ = 0.34, CI (0.25, 0.48), *p* < 0.001, *n* = 1162. |
| Fergusson et al. (2006)  New Zealand | RCT | No significant differences in physical IPV between intervention and control groups.  36m (mother report):  Physical IPV: χ^2^ = NR, *p* = 0.60, *n* = 391. |
| Fergusson et al. (2013)  New Zealand | RCT | No significant differences in mother reported IPV (aggregated psychological and physical) victimisation scores or father reported IPV perpetration scores between intervention and control groups. 5y and 9y (pooled effect):  IPV total mother report: *d^^^* = NR, *p* > 0.60, *n* = 370; IPV total father report: *d^^^* = NR, *p* > 0.80, *n* = 370. |
| Heyman et al. (2019)  USA | RCT | No significant differences in psychological or physical IPV between the intervention and control groups at any follow-up.  8m (mother report):  Psychological IPV: *d* = -0.10, CI (-0.36, 0.16), NS, *n* = 368; Physical IPV: OR = 0.83, CI (0.47, 1.45), NS, *n* = 368.  15m (mother report):  Psychological IPV: *d* = -0.10, CI (-0.39, 0.19), NS, *n* = 368; Physical IPV: OR = 0.80, CI (0.42, 1.52), NS, *n* = 368.  24m (mother report):  Psychological IPV: *d* = 0.01, CI (-0.25, 0.27), NS, *n* = 368; Physical IPV: OR = 1.25, CI (0.68, 2.29), NS, *n* = 368. |
| Heyman et al. (2020)  USA | RM | No significant differences in psychological or physical IPV from baseline to immediately post-intervention and follow-up.  Immediate (aggregated mother and father report):  Moderate psychological IPV: *g* = -0.02, NS, *n* = 171; Severe psychological IPV: *g* = -0.02, NS, *n* = 171; Moderate physical IPV: *g* = -0.06, NS, *n* = 171; Severe physical IPV: *g* = -0.12, NS, *n* = 171.  7m (aggregated mother and father report):  Moderate psychological IPV: *g* = -0.06, NS, *n* = 122; Severe psychological IPV: *g* = -0.06, NS, *n* = 122; Moderate physical IPV: *g* = -0.01, NS, *n* = 122; Severe physical IPV: *g* = -0.08, NS, *n* = 122. |
| Jensen et al. (2021)  Rwanda | Cluster RCT | Significantly greater decrease in physical and sexual IPV from baseline to immediately post-exposure and to follow-up in the intervention group compared to the control group, as reported by mothers but not fathers.  Immediate:  Mother report: IRR^†^ = 0.616, CI (0.425, 0.893), *n* = 523;  Father report: IRR^†^ = 0.897, CI (0.708, 1.134), *n* = 450.  12m:  Mother report: IRR^†^ = 0.442, CI (0.238, 0.820), *n* = 523;  Father report: IRR^†^ = 0.842, CI (0.559, 1.226), *n* = 450. |
| Kan et al. (2014)  USA | RCT | No significant differences in psychological IPV between intervention and control groups at follow-up.  30m (aggregated mother and father report):  IPV: β = 0.50, SE = 2.55, CI (-4.51, 5.51), *p* = 0.846, *n* = 139 (sourced from author correspondence). |
| McConnell et al. (2017)  UK | RM | Significant reduction from pre-intervention to immediate post-intervention and/or 6m post intervention for: father reported overall controlling behaviour and mother reported total IPV, emotional abuse, violence, injury and denial minimization, but not intimidation, economic abuse, isolation, threat/coercion, sexual abuse, or using children.  Immediate:  Father report:  Overall controlling behaviour: *z =* 3.145, *p* = 0.002, *n* = 38.  Mother report:  Total score: *z=*-2.295, *p* = 0.022, *n* = 20; Emotional abuse: *z =* **-**2.588, *p* = 0.010, *n* = 20; Violence: *z =* -2.023, *p* = 0.043, *n* = 20; Injury, denial minimisation, intimidation, economic abuse, isolation, threat/coercion, sexual abuse, using children: NS, *n* = 18 to 20.  6m:  Father report:  Overall controlling behaviour: *z =* 3.507, *p* < 0.001, *n* = 38;  Mother report:  Total score: *z =* -3.099, *p* = 0.002, *n* = 20; Emotional abuse: *z =* -2.630, *p* = 0.009, *n* = 20;  Violence: *z =* -2.201, *p* = 0.028, *n* = 20; Injury: *z =* -2.203, *p* = 0.043, *n* = 19; Denial minimisation: *z =* -2.295, *p* = 0.022, *n* = 20; Intimidation, economic abuse, isolation, threat/coercion, sexual abuse, using children: NS, *n* = 18 to 20. |
| Rhoades (2015)  USA | RCT | Significantly lower psychological IPV, but not physical or severe physical IPV, at 12m follow-up in intervention compared to control group. No significant differences in psychological, physical or severe physical IPV at 30m follow-up.  12m (mother report):  Psychological IPV: *d* = -0.12, *p* < 0.05, *n* = NR; Physical IPV: χ^2^ = NR, NS, *n* = NR; Severe physical IPV: χ^2^ = NR, NS, *n* = NR.  30m (mother report):  Psychological IPV: *d* = -0.06, NS, *n* = NR; Physical IPV: χ^2^ = NR, NS, *n* = NR. |
| Setodeh et al. (2019)  Iran | RCT | Significantly lower social, sexual and overall IPV in the intervention group compared to the control group immediately post exposure. No significant differences for psychological, physical or financial IPV.  Immediate (mother report):  Social IPV: *t = NR, p* = 0.02, *n* = 150; Sexual IPV: *t = NR, p* = 0.04, *n* = 150; Overall IPV: *t = NR, p* = 0.03, *n* = 150;  Psychological, physical and financial IPV: *t = NR, p* > 0.05, *n* = 150. |
| Stover (2015)  USA | RT | Significantly lower IPV post-exposure compared to pre-exposure across both intervention groups (Fathers for Change (F4C) and individual drug counselling). No significant difference in reduction in physical aggression between the intervention groups. Immediate and 3m (father report):  *F*(time) = 3.73, *p* = 0.035, *n* = 18.  *F*(group x time) = 1.23, NS, *n* = 18. |
| Stover et al. (2019)  USA | RT | Significant reduction in psychological and physical IPV in the Fathers for Change (F4C) parenting intervention group. Significant reduction in total, psychological and physical IPV in the alternative parenting intervention group. No significant differences in reduction in scores between F4C group and alternative parenting intervention group.  3m (father report):  Total IPV:  *z_between (intercept)_* = -0.74, NS, *n* = 62; *z_between (slope)_*  = 1.48, NS, *n* = 62; *z_within F4C_* = -0.26, NS, *n* = 62; *z_within alternative parenting intervention_* = -1.85, *p* < 0.05, *n* = 62. Psychological IPV:  *z_between (intercept)_* = 1.35, NS, *n* = 62; *z_between (change)_*  = -0.38, NS, *n* = 62; *z_within F4C_* = -2.66, p < 0.001, *n* = 62;*z_within alternative program_* = -2.35, *p* < 0.01, *n* = 62; Physical IPV:  *z_between (intercept)_* = 0.96, NS, *n* = 62;*z_between (change)_*  = 0.78, NS, *n* = 62; *z_within F4C_* = -1.68, *p* < 0.05, *n* = 62; *z_within alternative program_* = -2.77, *p* < 0.001, *n* = 62. |
| Stover et al. (2020)  USA | Repeated measures | Significant reduction in scores from baseline to immediately post-exposure.  Immediate (mother report):  Program non-completers: *z =* -3.07, *p* < 0.05*, r* = 0.26, *n* = 101;  Program completers: *z =* -9.29, *p* < 0.01*, r* = 0.45, *n* = 272. |

*Note.* Where change score (pre-to post- exposure) comparisons between the intervention and control group were available those are reported here. If unavailable, comparisons between post-exposure scores for the intervention and control groups are reported. If unavailable, comparisons between pre- and post-exposure score for the intervention group are reported. Adjusted effects were prioritised over unadjusted effects where available.
IPV = Intimate partner violence; CT = Controlled trial, RCT = Randomised controlled trial, RM = Repeated measures; RT = Randomised trial
m = month(s), w = week(s), y = year(s); CI = 95% Confidence intervals, *d* = Cohen’s *d,* g = Hedges *g*, IRR = Incidence rate ratio of interaction between group assignment and pre-post test scores, OR = Odds ratio, SE = Standard error, t = t-test statistic, z = z-score, χ^2^ = Chi-square, β = Standardised beta, ^†^ = denotes adjusted effect, ^^^ = denotes statistic derived from Generalised Estimating Equation model, NR = not reported, NS = not significant.
